# Right atrial cardiac lipoma with distinctive imaging characteristics. A rare case report and literature review

**DOI:** 10.3389/fcvm.2022.1043427

**Published:** 2022-12-01

**Authors:** Qiqing Chen, Dayan Yang, Lili Liu, Xiangxiang Jing

**Affiliations:** Department of Ultrasonography, Hainan General Hospital (Hainan Affiliated Hospital of Hainan Medical University), Haikou, China

**Keywords:** contrast-enhanced ultrasonography, transthoracic echocardiography, diagnosis, cardiac lipomas, multimodal imaging

## Abstract

Cardiac lipomas are rare primary cardiac tumors that are often only detected incidentally during other examinations. Lipomas of the right atrium are particularly rare. In this report, we describe the case of a patient presenting with a mixed cystic-solid lipoma in the right atrium. The symptoms, imaging findings, and treatment strategies associated with this case are discussed herein. This 65-year-old female patient reported to our hospital due to exertional chest tightness, shortness of breath, and occasional chest pain for over 1 year. She subsequently underwent transthoracic echocardiography and contrast-enhanced ultrasonography, both of which revealed a cystic-solid mass in the right atrium. The transthoracic computed tomography scan showed a dense patchy shadow in the right atrium. The mass was completely excised from the atrial septum, and subsequent histopathological examination confirmed its identity as a lipoma. Surgical resection remains the primary treatment approach for cardiac lipomas, and multimodal imaging is of key importance for the diagnosis and follow-up monitoring of affected patients.

## Introduction

Cardiac lipomas are rare primary cardiac tumors, the majority of which are asymptomatic (1). Clinical manifestations in affected patients generally vary as a function of tumor size and location, with some patients experiencing symptoms attributable to the compression of heart chambers by a large tumor volume or the involvement of the myocardium or heart valves that ultimately lead to their presentation for symptom. Transthoracic echocardiography remains the significant imaging approach when diagnosing cardiac tumors. Cardiac lipomas are rare, and largely present as solid, sessile masses with a soft texture and deformable shape that varies with the cardiac cycle upon imaging examination. Here, we describe a rare case report of a cystic-solid lipoma of the right atrium that was pathologically diagnosed as a lipoma following surgical excision. In addition to evaluating two-dimensional transthoracic echocardiography (2D-TTE), contrast-enhanced ultrasound (CEUS) and transthoracic computed tomography (CT), we additional discuss our experiences and associated surgical and pathological findings.

## Case report

### Medical history and physical examination

A 65-year-old female presented to the hospital with chest tightness and dyspnea that had been present for more than 1 year and were aggravated by exercise, co-occurring with dull, non-radiating chest pain that was relieved following rest for about 20 min. Transthoracic echocardiography conducted in another hospital revealed a space-occupying lesion, leading to her presentation to our hospital for additional treatment. On physical examination, the patient exhibited a heart rate of 76 bpm and a blood pressure of 128/85 mmHg. She had no history of diabetes, hypertension, or coronary heart disease. Specialist examination did not reveal any abnormal uplift in the precordial area nor any abnormalities in the valve areas, although slightly enlarged cardiac dullness was detected. No obvious murmur was heard in each valve area. Electrocardiography results revealed sinus rhythm with low voltage of the limb leads. Routine hematological, biochemical, and thyroid function test results were normal.

### Echocardiography examination

**FIGURE 1:**
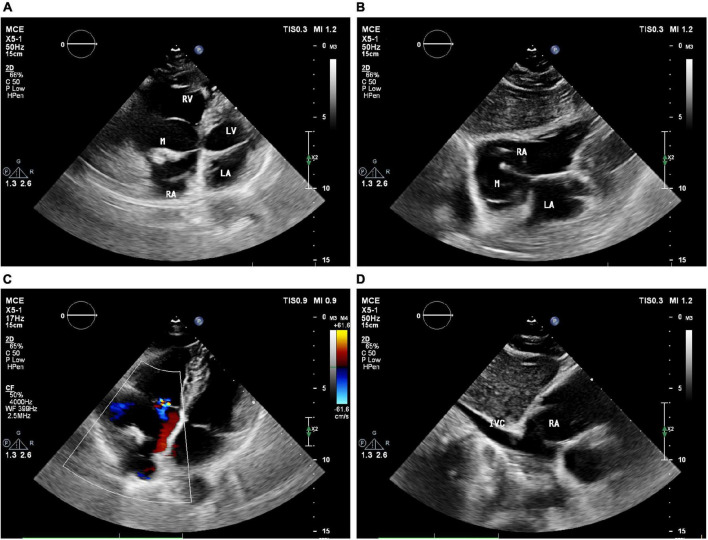
Echocardiography showed a large cystic-solid mixed echogenic lesion in the right atrium. Apical four-chamberview **(A)** and subxiphoid four-chamber view **(B)** showed that the mass was attached to the right atrial surface of the atrial septum. Ultrasound findings of tricuspid valve orifice **(C)** andinferior vena cava **(D)**. M = (intracardiac mass).

Two-dimensional transthoracic echocardiography (2D-TTE, Philips EPIQ CVx-Philips Medical Systems, Andover, MA, USA) revealed mild dilatation of the right atrium, with a predominantly cystic-solid mixed echogenic lesion that was present the right atrium with a rounded shape and a pedicle attached to the right atrium (Figures 1A, B). The atrial septum measured approximately 40 mm × 44 mm proximal to the fossa ovalis on the side of the right atrium, and significant echoic enhancement was observed for the solid portion of this mass (about 30 mm × 15 mm in size). No apparent obstruction or hemodynamic abnormalities were observed in the Valvular orifice (Figure 1C). No obvious abnormalities were apparent upon sonogram-based examination of the inferior vena cava (Figure 1D). Echocardiography confirmed the presence of a right atrial cystic solid mixed echogenic space-occupying lesion of uncertain etiology that was primarily cystic, with significantly enhanced echoic signal in the solid regions.

### Contrast-enhanced ultrasonography

**FIGURE 2:**
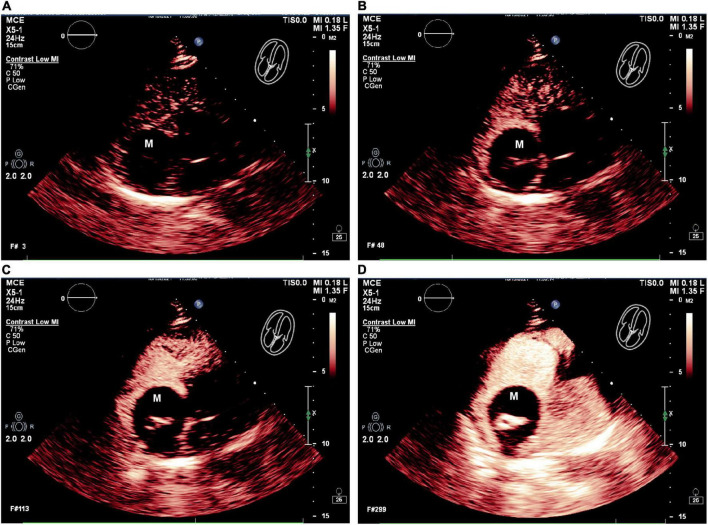
The RA mass was seen on contrast enhancement ultrasound (CEUS). **(A–D)** CEUS results showed that in different cardiac cycles, which the solid echo mass in the right atrium tumor revealed high contrast enhancement, and no contrast enhancement is evident in the surrounding anechoic area. M = (intracardiac mass).

The patient next underwent contrast-enhanced ultrasonography (Philips EPIQ CVx-Philips Medical Systems, Andover, MA, USA). After initiating the intracardiac contrast mode, the depth, gain, and instrument frame rate were adjusted such that the target lesions were clearly visible, after which the mechanical index (MI) was set to 1.0. Then, using SonoVue (Bracco, Italy) as an acoustic contrast agent, 5 mL of 0.9% sodium chloride was mixed with SonoVue microbubbles, and 2.4 mL of this mixture was gradually injected *via* the patient’s left cubital vein to observe the filling of the cardiac cavity, the lesion site, and myocardial tissue contrast-enhanced performance. The resultant dynamic image was stored for analysis. CEUS results revealed high contrast enhancement in the solid echoic mass in the center of the right atrial tumor, while no contrast enhancement was evident in the surrounding anechoic area (Figure 2). No abnormal contrast enhancement was observed in the other cardiac chambers or the myocardium. Accordingly, this right atrial cystic-solid mixed space-occupying lesion was tentatively diagnosed as a potential cardiac teratoma.

### Transthoracic computed tomography

**FIGURE 3:**
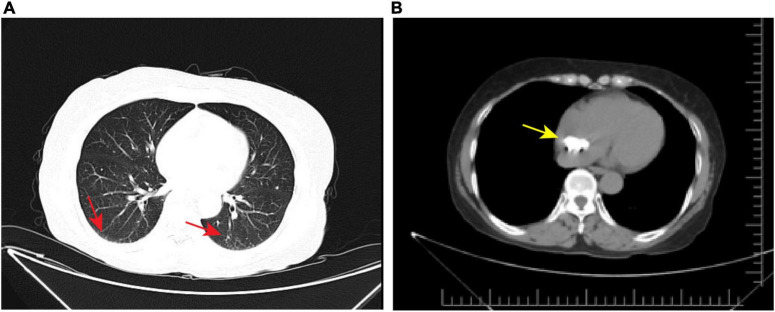
Axial view of chest computed tomography. **(A)** Chest CT showed low levels of inflammation in both lungs (red arrow). **(B)** Chest CT also showed a dense patchy shadow in the RA (yellow arrow).

The patient subsequently underwent chest CT examination, which showed low levels of inflammation in the middle lobe of the right lung, the lower lingual segment of the upper lobe of the left lung, and the lower lobes of both lungs (Figure 3A). In addition, the chest CT scan also exhibited a dense patchy shadow in the right atrium (about 26 mm × 14 mm in size) (Figure 3B). When measuring the lesion area in Hounsfield Units (HU), it exhibited a density of 394.6 HU, which was significantly higher than the density of normal fatty tissue. Although further conclusions regarding the etiology of this tumor could not be drawn based on these findings.

### Surgical and pathological findings

**FIGURE 4:**
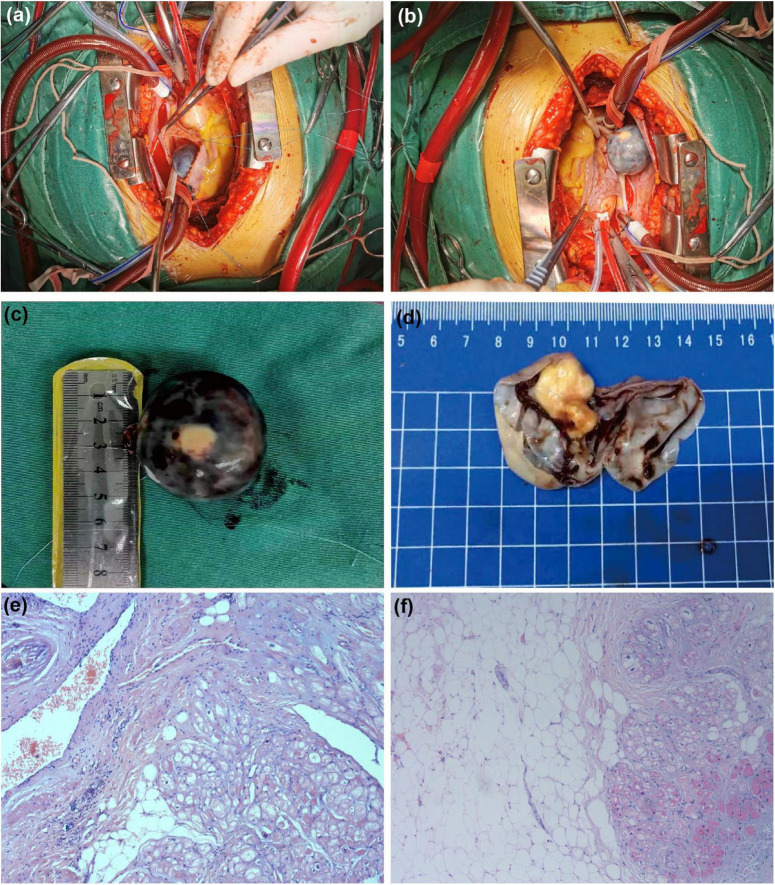
Surgical and pathological results. The tumor was spherical mass **(A)** and that was connected to the right atrial side of the right atrial septum under the oval fossa with a small tumor pedicle **(B)**. **(C)** The *ex vivo* tumor was black spherical. **(D)** It can be observed that the yellowish white solid tumor with rough texture is attached to one side of the cyst wall after dissecting the tumor. **(E,F)** Stained with hematoxylin and eosin (H&E) at 10 magnification: the tumor comprised mature adipocytes with entrapped myocardial cells.

After the above examinations had been completed, the patient underwent elective right atrial tumor resection. Intraoperative examination revealed moderate cardiac enlargement, and a spherical mass with a size of about 3.0 cm × 4.0 cm could be seen in the right atrium, the tumor was connected to the right atrial side of the right atrial septum under the oval fossa with a small tumor pedicle (Figures 4A, B). Subsequent dissection thereof resulted in the outflow of a large volume of dark red liquid, after which a solid yellow-white tumor with a rough texture was observed attached to one side of the cyst wall (Figures 4C, D). Postoperative pathological examination *via* light microscopy revealed large sheets of mature adipocytes interspersed between the myocardium, leading to the diagnosis of a cardiac lipoma (Figures 4E, F).

### Follow-up

Seven days after tumor excision, TTE revealed a normal chamber size and appropriate wall motion, with only limited levels of physiologic regurgitation of the tricuspid valve, and a left ventricular ejection fraction of 60%.

## Discussion

Cardiac lipomas are rare, comprising just 2.4% of all benign cardiac tumors (2). These masses are histologically similar to lipomas detected in other parts of the body, and originate from the mesoderm. Roughly 50, 25, and 25% of cardiac lipomas are of subendocardial, subepicardial, and myocardial origin, respectively (3). As most patients harboring cardiac lipomas do not experience any symptoms, these masses are often only detected incidentally (4). When patients do experience symptoms, they are directly associated with tumor size and location, with myocardial tumors impacting the cardiac conduction system, potentially causing intractable arrhythmias. In contrast, myocardial involvement can lead to conduction disturbances and other forms of arrhythmia including premature beats or atrial fibrillation. When large tumors are present within the heart cavities, this can result in abnormal cardiac hemodynamics, while heart valve involvement can result in valvular insufficiency or stenosis and associated clinical symptoms.

Transthoracic echocardiography (TTE) can provide detailed insight regarding the size, shape, location, activity, and secondary hemodynamic changes associated with a given tumor (5). As TTE is non-invasive, repeatable, and does not expose patients to any radiation, it is the imaging modality of choice. CEUS can offer insight regarding tumor vascularity. Cardiac lipomas generally contain relatively few blood vessels, while extensive vascularization may be indicative of malignancy. Surgical resection is the primary approach used to treat cardiac lipomas.

Literature reports suggest that cardiac lipomas are generally round, homogeneous masses with an internal echoic signal and performance that is associated with their localization (6). Intracardiac lipomas are generally hyperechoic and may have capsules, whereas pericardial lipomas are often poorly-defined hypoechoic masses with morphological characteristics that vary with the cardiac cycle. TTE can enable the further establishment of tumor location and the involvement of valves and surrounding tissue structures, while CEUS largely reveals limited contrast agent perfusion (4). The ultrasonographic findings observed in the present case are distinct from those reported previously in cases of cardiac lipoma. In this patient, TTE revealed a round cystic-solid space-occupying lesion with a clear boundary that did not deform with the cardiac cycle. Contrast echocardiography of the left heart further exhibited the presence of a cystic-solid mixed heterogeneous space-occupying lesion in the right atrium, with a solid echoic mass within the lesion exhibiting contrast agent hyperperfusion whereas no contrast agent perfusion was evident in the surrounding anechoic area. These imaging results are likely to result in the preliminary misdiagnosis of this mass as a cardiac teratoma.

The confluence of several factors led to the initial misdiagnosis of the present case, and it is important that these factors be reviewed to guide the evaluation of patients exhibiting similar clinical manifestations. Firstly, this case exhibited 2D-TTE findings distinct from those typically associated with cardiac lipoma cases, as prior literature reports suggest that cardiac lipomas often appear as solid hyperechoic masses with an uneven signal and a clear boundary that deforms with the cardiac cycle (4). In this case, however, we observed a mixed cystic-solid mass that did not deform with the cardiac cycle. We are not aware of any similar reports in the relevant literature, which is summarized in Table 1 (2, 4, 7–10). Secondly, common cardiac lipomas generally exhibit low levels of contrast enhancement upon CEUS examination. In this case, however, CEUS revealed high levels of contrast enhancement in the solid region of this mass together with an absence of any contrast enhancement in the anechoic region. Thirdly, cardiac teratomas are a relatively rare form of cardiac tumor and are more common in pediatric patients (11, 12), comprising ∼1% of all cardiac tumors in adults. Cardiac teratomas may present as polycystic mixed echoic masses, with some cysts being filled with hyperechoic components including fat or bone. Reported ultrasonographic findings associated with cardiac teratomas are similar to those observed in the present case, accounting for the initial misdiagnosis of this patient. However, pathological examination was ultimately able to provide a more accurate definitive diagnosis in this case.

**TABLE 1:** Literature review of cardiac lipoma case reports.

Case reports	Location	Size of lipoma	TTE	CEUS	Management
Fang (4)	RV	4.0 x 1.6 cm	hyperechoic solid mass	Slight enhancement	Surgical
Cao (8)	LV	5.0 × 3.0 cm	solid mass	no enhancement	Surgical
Dan et al. (10)	IVS	3.6 × 2.0 cm	homogeneous parenchymal mass	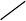	Surgical
Hsiao et al. (9)	pericardium	6.2 × 2.5 cm	homogenous large mas	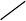	Surgical
Arı (2)	LV	2.1 × 1.6 cm	solid mass	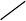	Surgical
Li (7)	LV wall	1.9 × 1.4 × 1.2 cm	irregular echo dense mass	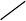	Surgical

RV, right ventricular; LV, left ventricular; IVS, interventricular septum.

It is important to differentiate between cardiac lipomas, such as those observed in the present case, and several other diseases. Cardiac liposarcomas are extremely rare and account for only ∼1% of all primary cardiac malignancies (13–16). Affected patients may experience symptoms including chest pain, tightness, and difficulty breathing at an earlier time point, and they frequently present with tumors that have already undergone distant metastasis. 2D-TTE examination of cardiac liposarcomas generally reveals an irregularly shaped heterogeneous hyperechoic mass, with larger tumors potentially harboring necrotic regions. On CEUS examination, cardiac liposarcomas typically exhibit heterogeneous regions of high enhancement, with no enhancement in necrotic areas. Well-differentiated liposarcomas are often indistinguishable from cardiac lipomas on 2D-TTE examination, leading to the potential for misdiagnosis such that only pathological examination can facilitate accurate identification. Cardiac metastases, which most frequently arise from primary tumors of the liver (17), may also exhibit a presentation similar to that of cardiac lipomas. The most frequently involved areas include the right heart and pericardium. These metastases are typically irregularly shaped, with a wide base, little activity, heterogeneous echoic signal, and evidence of refractory pericardial effusion. CEUS examination may reveal internal inhomogeneous hyperenhancement and hypervascular masses. Cardiac myxomas are lobulated structures with low-to-moderate echoic signal and low contrast enhancement on CEUS examination that usually occur at the fossa ovalis of the interatrial septum. Cardiac lymphomas are primarily hypoechoic or slightly hyperechoic, with unclear borders, uneven echoic signal, lower levels of liquefaction and calcification, and an irregular “cauliflower-like” shape (18). Varying degrees of secondary pericardial effusion may be evident in cases of pericardial involvement. When this disease is more advanced, adjacent tissues and blood vessels are frequently affected. CEUS examination generally exhibits high levels of contrast enhancement. Atrial thrombus formation may also mimic some of the characteristics of cardiac lipomas, primarily occurring in patients with a history of rheumatic heart disease, atrial fibrillation, or venous thrombosis. Echocardiography reveals an irregularly shaped mass with a wide base and an inhomogeneous echoic signal that does not oscillate with the cardiac cycle. CEUS examination of atrial thrombi generally fails to reveal any contrast medium perfusion, and fresh thrombi can form with limited contrast medium perfusion.

## Conclusion

While some cardiac tumors are associated with obvious or distinctive ultrasonographic findings, others do not exhibit these obvious features such that they may be more difficult to identify or diagnose. As cardiac lipomas generally have an insidious onset, their diagnosis is largely reliant on imaging examinations. Echocardiography is of particular value when diagnosing cardiac masses, and left cardiac contrast echocardiography is particularly advantageous as a means of distinguishing between benign and malignant tumors. Contrast echocardiography is a safe and sensitive approach that can offer insight regarding the blood supply of a given lesion, thus supporting differential diagnosis efforts. In summary, the present case emphasizes the importance of considering clinical manifestations, laboratory test results, and multimodal imaging findings when diagnosing patients with cardiac lipomas or other cardiac tumors.

## Data availability statement

The original contributions presented in this study are included in the article/supplementary material, further inquiries can be directed to the corresponding author.

## Ethics statement

Written informed consent was obtained from the individual(s) for the publication of any potentially identifiable images or data included in this article.

## Author contributions

XJ contributed to the conception of the case report. QC contributed to writing the manuscript. DY and LL contributed to the clinical data collection. All authors contributed to the article and approved the submitted version.
